# Utilizing Gamification Effect through Kahoot in Remote Teaching of Immunology: Medical Students’ Perceptions

**DOI:** 10.30476/JAMP.2022.93731.1548

**Published:** 2022-07-01

**Authors:** JANARTHANI LOHITHARAJAH, PUNITHALINGAM YOUHASAN

**Affiliations:** 1 Department of Pathophysiology, Faculty of Health-Care Sciences, Eastern University, Sri Lanka; 2 Centre for Medical and Health Sciences Education, Faculty of Medical and Health Sciences, The University of Auckland, New Zealand; 3 Department of Medical Education & Research, Faculty of Health-Care Sciences, Eastern University, Sri Lanka

**Keywords:** Gamification, Online learning, Formative feedback, Immunology, Medical education

## Abstract

**Introduction:**

Gamification of learning is a novel pedagogical approach in education, and Kahoot is one of the game-based learning platforms widely used for formative assessments in real-time. This study aimed to explore the medical students’ perception of using Kahoot in remote learning.

**Methods:**

The mixed-method study was carried out among 72 medical students (in third-year) at Eastern University, Sri Lanka, following a formative assessment on immunology conducted via zoom video conferencing and Kahoot. The students’ perception was collected through a google form, which consisted of 13 statements with a 5-point Likert scale and an open-ended question. Descriptive statistics and Mann-Whitney test were computed using SPSS ver. 25. A content analysis was employed to interpret the qualitative statements.

**Results:**

The participants’ age ranges from 23-28 years with male-female ratio of 1:1.57. The majority of the students felt happy (73.6%) while playing Kahoot remotely and recommended it (84.7%) for formative assessment in future. The participants agreed that Kahoot increased the focus, understanding of the subject, helped retain knowledge, motivated them to learn, provided fun during learning, and kept them active throughout. The majority of the participants agreed that Kahoot was an effective tool for distance learning. Internet connectivity and switching between two applications were identified as difficulties while playing remote mode Kahoot quizzes.

**Conclusion:**

The online gaming platform Kahoot has a positive impact on learning immunology. Kahoot maintains its fun and enjoyable nature and motivates students to learn during remote teaching of immunology.

## Introduction

Maintaining the students’ attention during teaching-learning activities is a real challenge for health professional educators ( 1 ). Advancement in technology enables educators to use innovative approaches and modalities to overcome the challenge, and gamification is one such innovation. Gamification refers to the use of game design elements to traditionally non-game contexts, and it emerged a decade ago ( 2 , 3 ). The application of game design elements to education began with the idea of capturing significant attention and engagement of adult learners for a long period of time ( 4 ). 

In medical education, gamification strategies are implemented to teach different topics and specialities to different levels of trainees, including undergraduates, postgraduates and residents ( 5 - 11 ). Systematic reviews of gamification effects in the health professions education showed that gamification is a promising way to improve learning outcomes by strengthening learning behaviours and attitudes towards learning ( 3 , 12 ). 

Formative assessment is one of the indispensable attributes of education, where gamification strategies are widely used. Formative assessment enhances student learning through constructive feedback and prepares students for summative assessments ( 13 , 14 ). Technology-enabled assessment has its own benefits over paper-based assessments, such as improving motivation, promoting engagement, and enabling effective feedback and learner-centeredness ( 15 ). Kahoot is one of the emerging game-based learning platforms widely used for formative assessment. It is a free, real-time, game-based learning platform which is easy to use. The teacher needs to sign up on https://kahoot.com/ to create an account, while the students do not require an account to sign in or download the application. The teacher can create quizzes, surveys or discussions with an interface designed in English, and the generated game pin is the key for students to join the game. Students can enter into the game with their preferred nicknames that appear on the board, and background music plays throughout the game. The above features make the learning environment live and enhance active participation and motivation. Players (students) compete with each other and the top responders for each question based on the correct answer and how quick they respond are revealed and the overall winners are displayed on scoreboards at the end of the game. The Kahoot environment encourages the students to solve the problem faster. The Kahoot quizzes can be played on any device and any location; however, an uninterrupted internet connection is necessary, which is the main challenge for this application ( 16 ).

Literature suggests that Kahoot is relatively new to medical education and is being used as a teaching-learning tool in different disciplines, namely histology, pathology, pharmacology, microbiology and immunology since 2017. COVID-19 pandemic has flipped the face-to-face mode of educational delivery into online. The transition from on-campus learning causes a lack of attention, less engagement and isolation. Kahoot is a feasible and efficient e-learning tool that enhances learning via increasing students’ engagement and motivation for active participation during face-to-face classroom teaching ( 10 , 11 , 15 , 17 ). However, the Kahoot platform has been predominantly used and investigated in a face-face classroom. It is essential to investigate Kahoot’s gamification effect in the pure online medical education environment. Thus, this pilot study aims to use Kahoot as a formative assessment tool in remote teaching for the first time and explore the students’ perceptions.

## Methods

### Study setting and population

A mixed-methods study was carried out among third-year medical students of the Faculty of Health-Care Sciences (FHCS), Eastern University, Sri Lanka, following a formative assessment on immunology in August 2021. Bachelor of Medicine, Bachelor of Surgery (MBBS) curriculum of FHCS is organised into three phases; namely, phase I (Pre-clinical), phase II (Para-clinical) and phase III (Clinical) with numerous formative and continuous assessments and four summative assessments of learning and the duration of the MBBS program is ten semesters (5 years). Immunology is taught in the 5^th^ semester (3^rd^ year) during the para-clinical phase. A teacher administering the module is given the responsibility of designing the formative assessment of each module.

### Study procedure

Kahoot teacher account was created after free signing up in ‘https://kahoot.com/’. Kahoot quiz was created at the ‘https://create.kahoot.it’, consisting of ten multiple-choice questions with pictures, and each question being timed for 90 seconds. The students were informed regarding the Kahoot based formative assessment a week earlier, and options were given to use one or two devices for Zoom. The Kahoot operation steps were discussed through a WhatsApp group. A zoom meeting was scheduled to conduct the formative assessment, and the meeting link was sent to the students. Instruction on how to join the Kahoot game was given to the students at the beginning of Zoom meeting. The students were requested to join the Kahoot game by entering the game pin in ‘https://kahoot.it/’. The Kahoot (teacher’s) screen with game pin and questions was shared through Zoom. 

**Figure 1:**
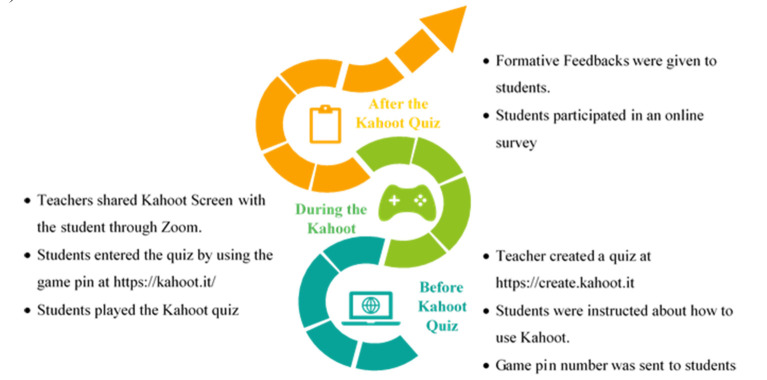
Procedure for playing Kahoot in remote mode

A batch of 3^rd^-year students who attended the quiz was invited to participate in this study. A total of 72 students consented and enrolled as convenience samples in the study. Data collection was done through an anonymous online survey, and the link of a google form was shared on the WhatsApp group immediately after the formative assessment ( Figure 1 ).

### Study instrument

The anonymous online survey consisted of seven sections for participants’ demography, prior usage of Kahoot, devices used, overall opinion, perceptions, likes and dislikes about Kahoot and comparison of real-time with the remote playing of Kahoot. The participants were requested to rate each of 13 perception statements using a 5-point Linkert scale ranging from 1 (strongly disagree) to 5 (strongly agree). A total of 10 perception statements were from the evaluation tool developed by Ismail and Mohammad ( 18 ). The items (statements) included in the questionnaire have been shown to have high internal consistency (Cronbach alpha=0.76) in a similar context ( 18 ). In addition, an open-ended question was included in the survey to identify the difficulties faced during Kahoot intervention by medical students. 

### Data analysis

The online anonymous survey data was retrieved in Microsoft Excel format from a google sheet, and then exported to SPSS Version 25 (IBM Corp., Armonk, NY, USA). Descriptive statistics were performed to present participants’ perceptions. Categorical data were presented in relative percentage (%) and counts (n). The median with interquartile range was used to describe the central tendency of the participants’ responses for each statement as the distribution was not normal. Mann-Whitney test was used to examine the association between genders on each survey item ( 18 ). An inductive content analysis was used to analyse the qualitative data from the open-ended question and categorise them into themes ( 19 ). 

### Ethical Consideration

The anonymous study was conducted as part of a teaching-learning activity to provide better students' learning experience, and this study was approved by the Ethics Committee of Eastern University, Sri Lanka. At the beginning of the teaching-learning activity, students were clearly informed that the voluntary submission of the online questionnaire indicated their consent to participate in the anonymized survey. Throughout the study, anonymity was maintained by treating the data with strict confidentiality, and participants' identifying information was not used in reporting the research.

## Results

### Demography of participants and type of devices used during the Kahoot quiz

A total of 72 students, whose age ranged from 23-28 years (mean=24.86: SD=1.25), participated in this study (response rate 98.6%). The majority were females (61.1%) and Sinhalese (59.7%). The participants represented 20 districts out of the 25 districts of Sri Lanka. Nearly 60% of the participants used two separate devices for Zoom and Kahoot during the formative assessment, and the majority used mobile phones (Table 1).

**Table 1:** Demographic data, type devices used during quiz and generic feedback

Variable	Subscale		Responses in % (n)
Gender	Male		38.9 (28)
	Female		61.1 (44)
Ethnicity	Sinhalese		59.7 (43)
	Tamils		30.6 (22)
	Muslims		9.7 (7)
Age	23-24		34.7 (25)
	25-26		61.1 (44)
	27-28		4.2 (3)
Type of devices used during Kahoot Quiz	I used the same mobile phone for zoom and Kahoot		31.9 (23)
	I used two different phones for zoom and Kahoot		29.2 (21)
	I used the same computer for zoom and Kahoot		8.3 (6)
	I used computer for zoom and phone for Kahoot		20.8 (15)
	I used phone for zoom and computer for Kahoot		9.7 (7)
Generic feedback	How fun was it?	Brilliant	19.4 (14)
		Really Good	34.7 (25)
		Good	40.3 (29)
		Not very good	4.2 (3)
		Awful	1.4 (1)
	Did you learn something?	Yes	95.8 (69)
		No	4.2 (3)
	How would you recommend it?	Strongly	26.4 (19)
		Recommend	58.3 (42)
		Undecided	8.3 (6)
		Slightly	2.8 (2)
		Not at all	4.2 (3)
	How did you feel?	Happy	73.6 (53)
		Normal	20.8 (15)
		Sad	5.6 (4)

### Students’ perception about Kahoot experience

This study revealed that 94.4% of the participants felt good about the virtual experience of Kahoot and 73.6% were happy while playing Kahoot remotely. More than 95% of the participants learned something from this experience, and 84.7% recommended using Kahoot in remote teaching/learning (Table 1). Moreover, all students’ perception statements regarding factors associated with learning, subject knowledge and feedback obtained an approximate median score of 04 (Table 2).

**Table 2:** Students’ perception about Kahoot experience

Statements	Median (IQR) for each item	Median (IQR)		z-Statistic	p
		Male	Female		
Kahoot helps me to focus on the subject.	4 (0)	4 (1)	4 (0)	-1.77	0.08
Kahoot motivates me to learn more.	4 (0)	4 (1)	4 (0)	-1.85	0.06
Learning with Kahoot is fun.	4 (0)	4 (0)	4 (0)	-0.57	0.57
Kahoot enhances my understanding of the subject.	4 (0)	4 (0)	4 (0)	-1.00	0.32
Kahoot helps to retain my knowledge.	4 (0)	4 (0)	4 (0)	-0.65	0.52
Kahoot simplifies the complex subjects.	4 (1)	4 (0)	4 (1)	-0.46	0.65
Kahoot facilitates my learning on the subject.	4 (0)	4 (0)	4 (0)	-0.02	0.98
Kahoot keeps me active throughout.	4 (0)	4 (0)	4 (1)	-0.78	0.44
Kahoot is an effective method to provide feedback.	4 (0)	4 (1)	4 (0)	-1.79	0.07
Kahoot is an effective method to correct my misconception on the subjects.	4 (0)	4 (0)	4 (0)	-0.60	0.55
Kahoot is an effective method for reflective learning.	4 (0)	4 (0)	4 (0)	-0.44	0.66
Kahoot is an effective method for distance learning.	4 (0)	4 (0)	4 (0)	-0.03	0.97
I can use Kahoot easily.	4 (0)	4 (0)	4 (0)	-0.01	0.99

IQR: Interquartile range

This indicated that Kahoot was an effective method for distance learning that enhanced focus, motivation, facilitation, active learning, and understanding of the subject, retaining knowledge and providing feedback to correct misconceptions. Mann-Whitney test showed that there was no significant median score difference (P>0.05) between females and males for each statement (Table 2).

### Difficulties faced by students

Thirty participants mentioned the difficulties of performing virtual Kahoot, which can be categorised into three themes: connection related (10/30), device-related (15/30) and application-related (05/30) (See Table 3). Insufficient internet connection (low speed) and switching between two applications were the main difficulties faced by the medical students. The themes and sample quotes are mentioned in Table 3.

**Table 3:** Students’ complaints about difficulties faced during Kahoot intervention

Type of Difficulty	Sample Quotes
Connection related	‘My answers were delayed some time because of low coverage, so my mark always was low.’
	‘When the Internet connection is slow it takes time for questions to appear and my response to be uploaded.’
	‘I couldn’t answer most of the questions; when Kahoot was loaded and connected on my browser, the time was over for answering most of the questions.’
	‘Slow. Can't submit answers.’
	‘I couldn’t really join the quiz because loading takes a lot of time.’
	‘Connection error.’
Device related	‘I faced difficulty during the assessment because I used a single device.’
	‘I used mobile for both apps, and while switching the apps, it kept reloading and I was disconnected every time. So, I couldn’t reveal my real performance.’
	‘It took a lot of time to load Kahoot, because I had to switch in between zoom and Kahoot to see questions.’
	‘Sometimes it takes a lot of time to change the tabs and sometimes it causes zoom to freeze. Those with two devices perform very quickly.’
	‘Going to both zoom and Kahoot with the same phone.’
	‘Using 2 devices was difficult’.
Application related	‘I don’t know how to click for answers”.
	‘I had no idea how to answer. So, I waited for questions to appear on the Kahoot screen too’.
	‘At the beginning I waited for questions to appear on Kahoot, so I missed 3 questions. I had no idea what I should do’.
	‘Sometimes we accidentally touch the wrong answer, and we can't go back and correct our answers’.
	‘I joined in the middle of the activity because the Kahoot app I downloaded was not the correct one, later I realized there was another way to join without downloading the application.
	‘For the first time it was difficult’.

## Discussion

Kahoot is a readily available game-based learning tool used in medical education to add liveliness, student engagement, and meta-cognitive supports with the limited requirements of instructor or student training. The gamified approach motivates the students to engage with their learning by flipping the classroom, empowering the learners, and enabling them to learn better than those who learn using traditional methods ( 18 ). Due to technological simplicity and free availability, Kahoot has been widely used in face-to-face classrooms in educational institutes, and it has been proved that Kahoot is an effective tool to motivate students to learn ( 10 , 11 , 15 , 17 , 18 , 20 - 23 ). This study showed the potential of Kahoot to enhance learning through a positive impact on motivation and engagement during remote teaching of immunology. 

Though in the current study, the participants experienced Kahoot for the first time, most of them enjoyed learning with Kahoot and recommended it for remote teaching/learning. Gaining students’ attention is one of the main elements in the learning process. Kahoot draws the attention of students through its colour, music and the excitement and encourages them to learn. This may also be a reason why Kahoot achieves the eLearning merits of student engagement ( 24 ).

In this study, most of the participants indicated that Kahoot kept them active throughout the session. In a virtual classroom, since students cannot see each other, they may feel lonely, and the teacher does not know how much students are involved with teaching-learning activities ( 25 ). When a student sees others’ (nick) names on the Kahoot screen, he or she feels connected with others in the classroom ( 20 ). The sound at each submission of the answer keeps the environment alive and gives the alarm to the teacher regarding students’ participation ( 26 ). 

The present study revealed that Kahoot is an effective method to provide feedback. The prevailing literature affirmed this notion as Kahoot is an effective tool to achieve the main aim of formative assessments of learning through constructive feedback. It provides immediate feedback after each question and enables the students to assess their level of knowledge and correct their misconceptions ( 15 , 27 ). The above feature is more helpful in a virtual classroom where it is hard to monitor each student ( 28 ). In the present study, nearly 85% of students were able to correct their misconceptions on a subject with the help of frequent feedback. The utilisation of Kahoot in the face-to-face classroom resulted in similar findings showing that Kahoot is an effective tool to provide feedback and correct students’ misconceptions regardless of the mode of teaching ( 11 , 15 , 18 ). 

The present study shows that the Kahoot approach creates a positive learning environment for the students in the aspects of fun, focus, motivation, facilitation, and active participation regardless of gender. The influence of gender in creating a motivational learning environment by gamification has been reflected differently in different studies. Erhel and Jamet (2013) reported that game-based learning creates motivational learning environments regardless of gender ( 21 ), while Ismail and Mohammad (2017) found that the male students were significantly more motivated by Kahoot than the female students ( 18 ). 

A quarter of the participants found that Kahoot cannot simplify the complex subject. This inability of Kahoot was noted in a cross-sectional study on first-year medical students of Universiti Sains Malaysia ( 18 ). However, a cross-sectional study following a formative assessment in pharmacology done in the FHCS, Eastern University, Sri Lanka, had a different finding in which more than 80% of the students mentioned that Kahoot simplifies the complex subjects ( 15 ). The differences in perceptions of this aspect are attributed to the subjects or topics or discipline regardless of the teaching mode. Having Kahoot based formative assessment for each topic enables the students to use this platform to simplify the complexity of the subject ( 15 , 27 ). 

Stable internet connection is the main challenge in using online game-based platforms, which was reported in this study. In a face-to-face classroom, students can see the questions on the teacher’s screen and use their device only to submit answers. Nevertheless, in a virtual classroom, students need to be online for both purposes. With an unstable or poor internet connection, switching between Zoom and Kahoot software is difficult for the students and interrupts their active participation. Since the Kahoot ranks the players according to the time taken to submit the answers, continuous internet connection is essential for active participation and motivation of students. The unstable internet connectivity and limited internet provision could apply to other developing nations and create a hindrance for developing online learning methods ( 29 ). The time duration of each question can be slightly increased if the quiz is used in a virtual classroom to compensate for the time lost during switching from one software to another. Most of the participants of this study had never used Kahoot before; some of them had a problem in clicking the answers. Difficulties in handling two applications and answering can be overcome by practice with more Kahoot quizzes conducted remotely.

This study could have limitations derived from the pilot character of the educational intervention. The study investigated Kahoot application among a particular batch of students with a limited sample size. Also, no explicit control group was defined and analysed in parallel. Thus, future studies are encouraged to compare education outcomes by Kahoot intervention versus traditional post-class assessments alone. In addition, qualitative research will likely be instructive in terms of enabling a deeper understanding of the phenomena.

## Conclusion

The online gaming platform Kahoot has a positive impact on learning immunology. This pilot study demonstrated that Kahoot maintains its fun and enjoyable nature and motivates the students to learn in remote teaching in immunology. The high acceptance from students indicates that Kahoot is a feasible and efficient formative assessment tool for remote teaching to increase students’ engagement and motivation for active participation. Further studies are recommended with Kahoot in different disciplines and subject areas to generalize the findings of this study to medical education. 

## Authors' contribution

J.L, P.Y contributed to the conception and design of the work; the acquisition, analysis, or interpretation of data for the work. All Authors contributed in drafting and revising the manuscript critically for important intellectual content. All authors have read and approved the final manuscript and agree to be accountable for all aspects of the work in ensuring that questions related to the accuracy or integrity of any part of the work are appropriately investigated and resolved.

## Conflict of Interest:

None declared.
